# Restoration of dysnatremia and acute kidney injury benefits outcomes of acute geriatric inpatients

**DOI:** 10.1038/s41598-021-99677-z

**Published:** 2021-10-11

**Authors:** Yu-Hsiang Chou, Feng-Ping Lu, Jen-Hau Chen, Chiung-Jung Wen, Kun-Pei Lin, Yi-Chun Chou, Meng-Chen Wu, Yung-Ming Chen

**Affiliations:** 1 Department of Internal Medicine, National Taiwan University Hospital, No.7, Chung Shan S. Rd., Taipei, 100225 Taiwan; 2 Department of Internal Medicine, College of Medicine, National Taiwan University, No. 1, Jen-Ai Road, Section 1, Taipei, 100233 Taiwan; 3 Department of Geriatrics and Gerontology, National Taiwan University Hospital, No.7, Chung Shan S. Rd., Taipei City, 100225 Taiwan

**Keywords:** Diseases, Medical research, Nephrology

## Abstract

Dysnatremia and dyskalemia are common problems in acutely hospitalized elderly patients. These disorders are associated with an increased risk of mortality and functional complications that often occur concomitantly with acute kidney injury in addition to multiple comorbidities. In a single-center prospective observational study, we recruited 401 acute geriatric inpatients. In-hospital outcomes included all-cause mortality, length of stay, and changes in functional status as determined by the Activities of Daily Living (ADL) scale, Eastern Cooperative Oncology Group (ECOG) performance, and Clinical Frailty Scale (CFS). The prevalence of dysnatremia alone, dyskalemia alone, and dysnatremia plus dyskalemia during initial hospitalization were 28.4%, 14.7% and 32.4%, respectively. Patients with electrolyte imbalance exhibited higher mortality rates and longer hospital stays than those without electrolyte imbalance. Those with initial dysnatremia, or dysnatremia plus dyskalemia were associated with worse ADL scores, ECOG performance and CFS scores at discharge. Subgroup analyses showed that resolution of dysnatremia was related to reduced mortality risk and improved CFS score, whereas recovery of renal function was associated with decreased mortality and better ECOG and CFS ratings. Our data suggest that restoration of initial dysnatremia and acute kidney injury during acute geriatric care may benefit in-hospital survival and functional status at discharge.

## Introduction

Disorders of serum sodium concentration, i.e., dysnatremia, including either hyponatremia or hypernatremia, are the most common electrolyte disturbance associated with morbidity and mortality in the elderly population^1–4^. A systematic review of the literature from 1966 to 2009 shows that the prevalence of hyponatremia increases from 15–30% for general hospitalized patients to as high as 50% or more for geriatric hospitalized patients^5^. Hospital-associated hyponatremia is a notable predictor of adverse patient outcomes including being discharged to a care home^6,7^. At the other end of the spectrum, hypernatremia occurs in 1–3% of hospitalized patients and is almost exclusively found in elderly individuals, critically ill patients, and nursing home residents^2,8,9^. Depending on the severity, clinical manifestations of dysnatremia may range from mild gastrointestinal upset and drowsiness to severe neurologic dysfunction such as seizure and coma^10^. Compared with patients with normonatremia, those with hyponatremia or hypernatremia were associated with longer hospital stays and a greater risk of mortality^3,11^. In addition to dysnatremia, serum potassium level disorders, i.e., dyskalemia, also occurred frequently in the elderly population. A retrospective survey from patients presenting to the emergency department found that the frequency of hypokalemia was 10–12% across all age groups, whereas hyperkalemia was observed increasingly from 0.8% in patients aged 16–21 years to 10.4% in those aged > 80 years^12^. Like dysnatremia, dyskalemia can negatively impact patient outcomes, and has been associated with increased in-hospital complications such as arrhythmic events and cardiac death^13,14^.

These electrolyte disorders, including dysnatremia or dyskalemia, not only impair patient outcomes directly, but also typically reflect the severity of underlying illnesses and comorbidities that tend to act in concert and lead to adverse consequences. In that sense, electrolyte imbalance may be considered a prognostic marker or even therapeutic target during acute patient care. Indeed, dysnatremia has been directly related to frailty and mortality in the National Health and Nutrition Examination Survey on community-dwelling adults^15^, the correction of which is associated with improved neurocognitive function and motor performance in hospitalized older patients^16,17^. Similarly, studies conducted in intensive care units have shown that early correction of dysnatremia or dyskalemia is associated with improved prognosis in critically ill patients^14,18^. However, not all investigators found a dependent relationship between correction of electrolyte imbalance and reduced mortality risk^19,20^, and information in this regard focusing on elderly inpatients is particularly lacking.

This prospective observational study aimed to investigate the impact of dysnatremia and/or dyskalemia on hospital outcomes in an acutely ill population of elderly patients admitted to a dedicated geriatric unit. Moreover, because the kidneys are the principal organs that maintain electrolyte homeostasis, we also searched for the prognostic implications of acute kidney injury (AKI) superimposed on concurrent electrolyte abnormalities.

## Results

### Baseline characteristics in patients stratified by electrolyte imbalance

Four hundred and ninety-three elderly patients admitted via the emergency room during the nine-month period were screened. 36 institutionalized residents and 56 readmitted patients were excluded due to complex chronic conditions or social issues. A total of 401 acute geriatric patients with a mean age of 84 years were enrolled. These patients were admitted to a single geriatric ward with the diagnosis of pneumonia with or without chronic obstructive pulmonary disease, urinary tract infection, cerebrovascular disease, cellulitis, and gastrointestinal bleeding, in descending order. The baseline clinical features are listed in Table 1. At admission, 212 (52.9%), 10 (2.5%), 46 (11.5%), and 55 **(**13.7%) patients exhibited hyponatremia, hypernatremia, hypokalemia, and hyperkalemia, respectively, and there existed a weak inverse correlation between serum sodium and potassium level (Supplementary Material Figure S2). During the initial 72 h of hospitalization, the overall prevalence of isolated dysnatremia (hypo- and hypernatremia), isolated dyskalemia (hypo- and hyperkalemia), and dysnatremia plus dyskalemia were 28.4%, 14.7% and 32.4%, respectively. Compared to patients without any imbalances, patients with dysnatremia and/or dyskalemia were more likely to have lower body mass index (BMI), higher Charlson comorbidity index (CCI), lower hemoglobin, higher proportions of pneumonia and peripheral arterial occlusive disease on admission, and a higher proportion using diuretics at discharge. Additionally, chronic kidney disease (CKD) was marginally more prevalent in patients with certain forms of electrolyte disorders (Table 1).

**Table 1:** Baseline characteristics of the study population with or without dysnatremia and dyskalemia.

	All (n = 401)	Dysnatremia plus dyskalemia (n = 130)	Dysnatremia alone (n = 114)		Dyskalemia alone (n = 59)		None (n = 98)			*P* value
Age, years (SD)	84 (8)	85 (8)	85 (9)		85 (8)		82 (8)			0.04
Male, n (%)	216 (54)	68 (52)	53 (47)		39 (66)		56 (57)			0.09
BMI (SD)	22.6 (4.4)	22.1 (4.7)	22.5 (3.8)		21.8 (3.9)		23.8 (4.7)			0.02
Diabetes mellitus, n (%)	114 (28)	42 (32)	27 (24)		19 (32)		26 (27)			0.04
Hypertension, n (%)	287 (72)	98 (76)	80 (70)		41 (70)		68 (69)			0.71
Hyperlipidemia, n (%)	133 (33)	44 (34)	37 (33)		15 (25)		37 (38)			0.46
CAD, n (%)	71 (18)	25 (19)	20 (18)		8 (14)		18 (18)			0.82
CHF, n (%)	74 (19)	24 (19)	23 (20)		15 (25)		12 (12)			0.2
PAOD, n (%)	29 (7)	11 (9)	4 (4)		11 (19)		3 (3)			< 0.01
CVA, n (%)	153 (38)	50 (39)	36 (32)		23 (39)		44 (45)			0.26
Dementia, n (%)	130 (32)	42 (32)	41 (36)		21 (36)		26 (27)			0.48
COPD, n (%)	76 (19)	22 (17)	26 (23)		10 (17)		18 (18)			0.65
CKD stage 4–5, n (%)	104 (26)	42 (32)	23 (20)		19 (32)		20 (20)			0.06
Malignancy, n (%)	21 (5)	10 (8)	4 (4)		5 (9)		2 (2)			0.14
Charlson comorbidity index (SD)	6.8 (2.5)	7.4 (2.6)	6.5 (2.1)		7.3 (2.8)		6.2 (2.3)			0.01
**Main diagnosis of admission**										
Pneumonia, n (%)	145 (36)	54 (42)	50 (44)		24 (41)		17 (17)			< 0.01
UTI, n (%)	93 (23)	27 (21)	30 (26)		13 (22)		23 (24)			0.78
Stroke, n (%)	33 (9)	4 (3)	3 (3)		5 (9)		21 (21)			< 0.01
Cellulitis, n (%)	27 (7)	10 (8)	11 (10)		2 (3)		4 (4)			0.27
GI bleeding, n (%)	9 (2)	2 (2)	2 (2)		1 (2)		4 (4)			0.57
Decompensated HF, n (%)	8 (2)	3 (2)	2 (2)		2 (3)		1 (1)			0.76
COPD exacerbation, n (%)	7 (2)	2 (2)	2 (2)		1 (2)		2 (2)			0.99
**Blood biochemistry data at admission**										
Hemoglobin, g/dl (SD)	11.6 (2.4)	11.4 (2.4)	11.5 (2.2)		11.1 (2.3)		12.4 (2.4)			< 0.01
White blood cell, K/µl (SD)	10.5 (5.0)	10.7 (5.0)	11.3 (5.0)		9.6 (4.1)		9.9 (5.5)			0.10
Creatinine, mg/dl (SD)	1.4 (1.2)	1.7 (1.6)	1.3 (0.9)		1.6 (0.9)		1.2 (0.7)			0.01
eGFR, ml/min/1.73 m^2^ (SD)	63 (35)	51 (33)	63 (37)		54 (34)		65 (32)			0.03
Sodium, mmol/L (SD)	133.1 (6.9)	131.0 (7.3)	129.8 (7.6)		136.9 (3.0)		137.6 (2.3)			< 0.01
Potassium, mmol/L (SD)	4.4 (1.0)	4.6 (1.4)	4.3 (0.4)		4.4 (1.3)		4.1 (0.4)			< 0.01
**Medication at discharge**										
Psychotropic agents, n (%)	154 (38)	49 (38)	50 (44)		22 (37)		33 (34)			0.49
RAS inhibitors, n (%)	98 (24)	30 (23)	28 (25)		14 (24)		26 (27)			0.94
Diuretics, n (%)	76 (19)	33 (25)	19 (17)		14 (24)		10 (10)			0.02
ESA, n (%)	4 (1)	4 (3)	0 (0)		0 (0)		0 (0)			< 0.01

*SD* standard deviation, *BMI* body mass index, *CAD* coronary artery disease, *CHF* congestive heart failure, *CKD* chronic kidney disease, *COPD* chronic obstructive pulmonary disease, *CVA* cerebrovascular accident, *RAS* renin angiotensin system, *GI* gastrointestinal, *GFR* glomerular filtration rate, *HF* heart failure, *PAOD* peripheral arterial occlusive disease, *UTI* urinary tract infection, *ESA* erythropoiesis-stimulating agent.

### Hospital outcomes in patients with or without electrolyte imbalance or AKI

**Figure 1:**
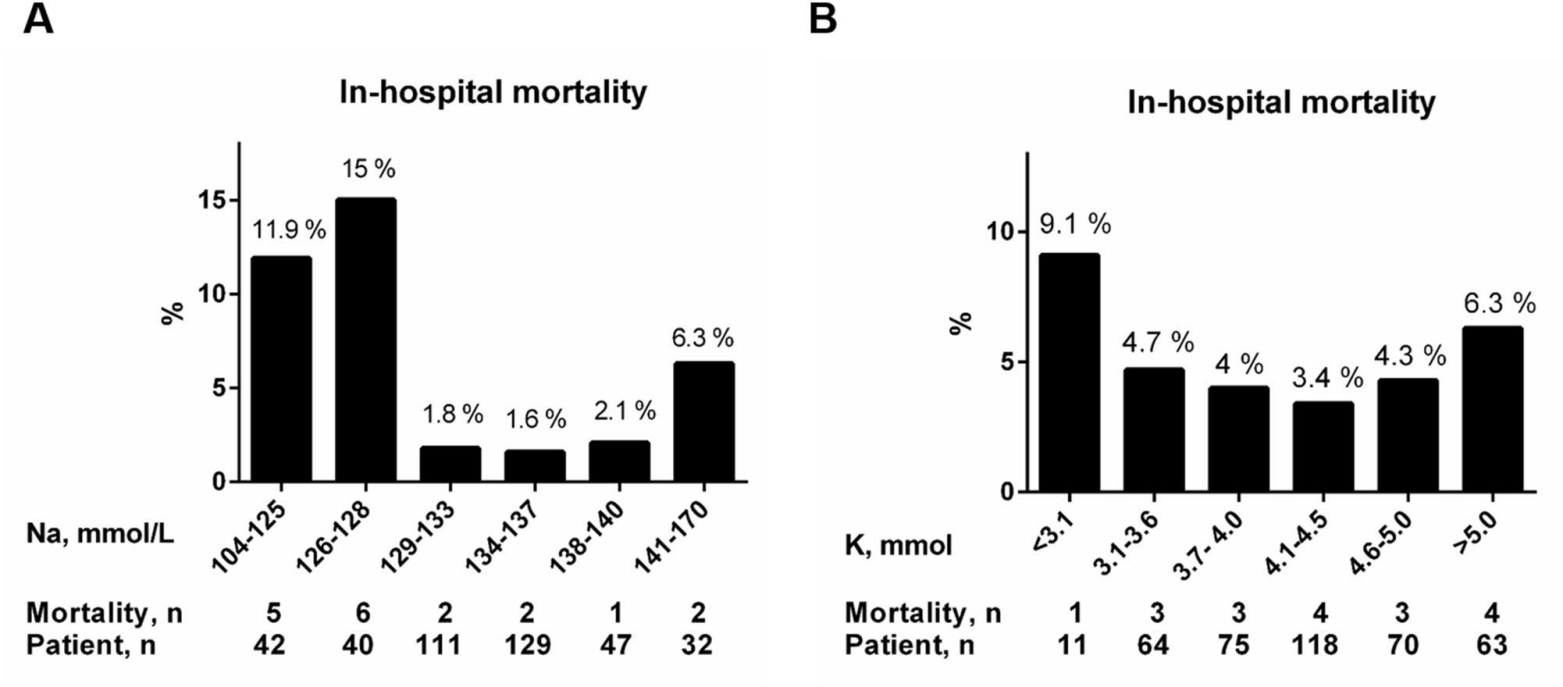
**(A,B)** The percentages of in-hospital mortality in patients with distinct categories of serum sodium and potassium levels at admission. Figures were created using the GraphPad Prism software (version 6.0).

**Figure 2:**
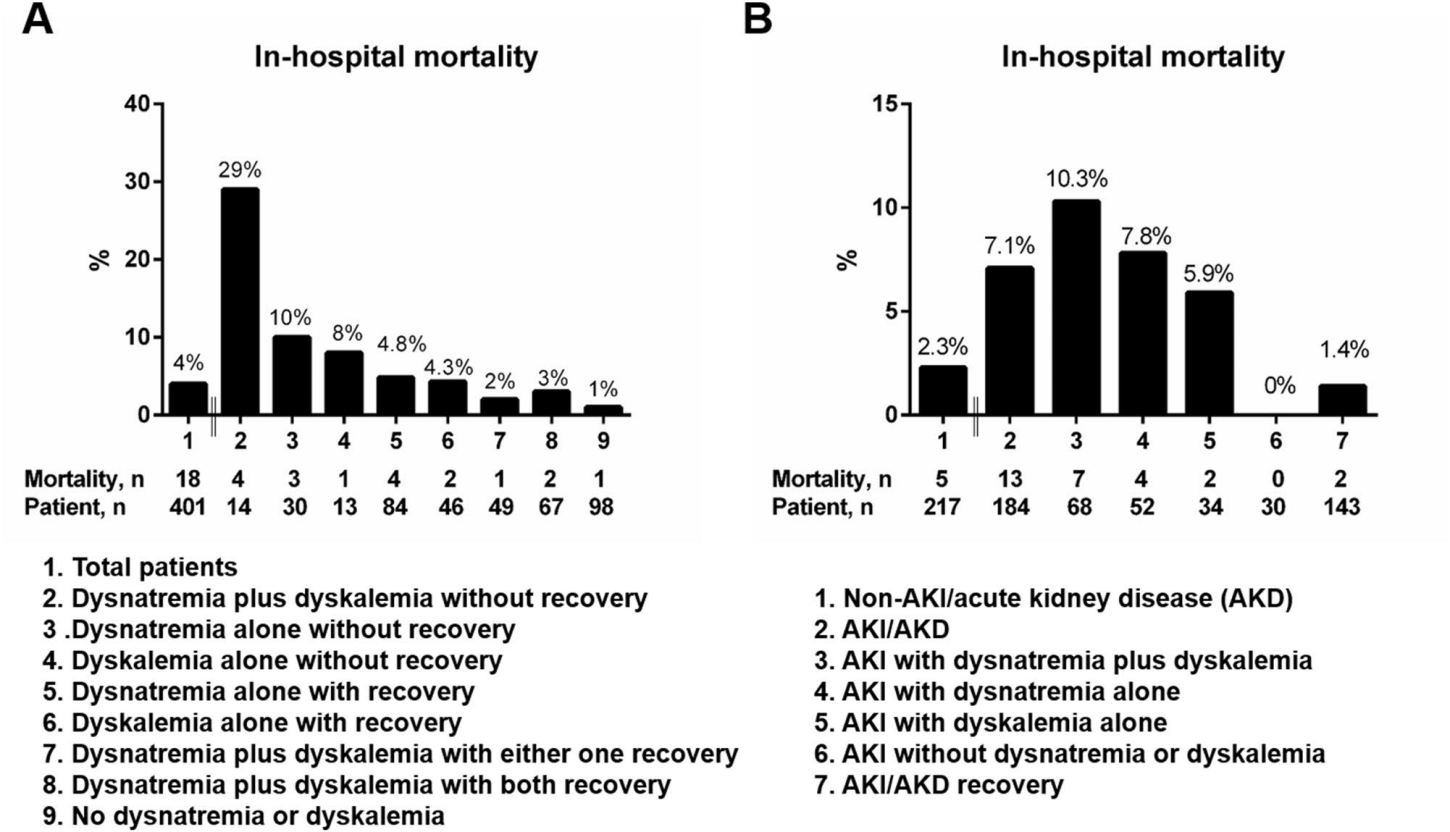
The percentages of in-hospital mortality in subgroups of patients with or without **(A)** development and resolution of electrolyte imbalances, and **(B)** development and recovery of AKI/acute kidney disease (AKD) in combination with electrolyte disorders. Figures were created using the GraphPad Prism software (version 6.0).

As shown in Supplementary Material Table S1, 184 (46%) of 401 patients met the designated criteria of AKI at admission or during initial hospitalization. This figure was significantly higher in patients with dysnatremia and/or dyskalemia than in those without any electrolyte imbalance. In contrast, no significant difference in the percentages of delirium was noted across the four subgroups. The overall recovery rates of dysnatremia, dyskalemia, and AKI were 72%, 73%, and 77%, respectively. Patients with dysnatremia plus dyskalemia exhibited the longest length of stay (LOS) (14.8 days, *P* < 0.01). The all-cause mortality rate was 4%, but there was no appreciable difference between groups. Further analyses showed a U-shaped association between mortality rates and serum sodium or potassium concentrations (Fig. 1). Patients with dysnatremia plus dyskalemia without restoration of imbalance displayed the highest mortality rate (29%) among others (Fig. 2A). Similarly, patients with AKI combined with electrolyte imbalances exhibited a substantially higher mortality rate than those with isolated AKI (Fig. 2B). Supplementary Material Figure S1 shows better functional outcomes and shorter LOS in patients with relatively normal serum sodium and potassium levels at admission.

With regard to changes in functional status (Table 2), patients with initial dysnatremia and/or dyskalemia displayed significantly worse ratings on the ADL scale, ECOG performance, and CFS, both upon admission and at discharge, compared with those without electrolyte imbalance. Of note, the percentages of ECOG improvement were appreciably lower in patients with electrolyte disorders compared with those without any imbalance.

**Table 2:** Functional status changes of the study population with or without dysnatremia and dyskalemia during hospitalization.

	All (n = 401)	Dysnatremia plus dyskalemia (n = 130)	Dysnatremia alone (n = 114)	Dyskalemia alone (n = 59)	None (n = 98)	*P* value
ADL score at admission* (SD)	42.7 (36.7)	35.5 (35.5)	38.3 (37.1)	42.3 (35.6)	56.0 (35.1)	< 0.01
ADL score at discharge* (SD)	45.4 (37.4)	39.3 (36.5)	40.0 (38.4)	44.9 (35.3)	60.3 (35.2)	< 0.01
ΔADL (SD)	3.0 (11.7)	4.3 (11.9)	1.8 (9.8)	0.3 (15.4)	4.3 (10.5)	0.08
ADL improvement, n (%)	154 (39.2)	45 (35.2)	38 (33.9)	23 (39.7)	48 (50.5)	0.06
ECOG at admission* (SD)	2.5 (1.2)	2.7 (1.2)	2.6 (1.3)	2.6 (1.2)	2.2 (1.2)	0.01
ECOG at discharge* (SD)	2.7 (1.2)	3.0 (1.1)	2.8 (1.3)	2.9 (1.2)	2.2 (1.2)	< 0.01
ΔECOG (SD)	0.2 (0.6)	0.3 (0.5)	0.2 (0.7)	0.2 (0.7)	0.1 (0.3)	0.10
ECOG improvement, n (%)*	144 (36)	35 (26.9)	41 (36.0)	18 (31.0)	50 (51.0)	< 0.01
CFS at admission* (SD)	5.6 (1.9)	5.9 (1.8)	5.8 (1.9)	6.0 (1.7)	5.0 (1.9)	< 0.01
CFS at discharge* (SD)	5.9 (2.0)	6.2 (1.9)	6.1 (2.1)	6.2 (1.9)	5.2 (1.8)	< 0.01
ΔCFS (SD)	0.3 (0.9)	0.4 (0.8)	0.3 (1.1)	0.3 (0.9)	0.2 (0.5)	0.37
CFS improvement, n (%)	51 (12.7)	14 (10.8)	15 (13.2)	4 (6.8)	18 (18.4)	0.16

*ADL* activities of daily living, *SD* standard deviation, *ECOG* eastern cooperative oncology group, *CFS* clinical frailty score.

*P < 0.05.

### Effects of resolution of electrolyte imbalance and AKI on outcomes

As shown in Table 3 (model 1), patients with isolated dysnatremia or dysnatremia plus dyskalemia, displayed worse ratings on the ADL scale, ECOG performance and CFS at discharge compared to those without any imbalance. Similarly, patients with isolated dyskalemia or dysnatremia plus dyskalemia exhibited longer LOS. In addition, AKI development was associated with a higher risk of in-hospital death (odds ratio, 9.16; 95% confidence interval, 1.12 to 74.9). Delirium during hospitalization was associated with worse ratings on the ADL scale, ECOG performance and CFS. Of note, diuretic use was associated with a better rating of ECOG performance.

**Table 3:** Multivariate regression analyses of the effects of electrolyte imbalance and AKI on in-hospital complications and functional status at discharge.

	Mortality		LOS		Functional scores at discharge					
					ADL		ECOG		CFS	
	OR	95% CI	β	95% CI	β	95% CI	β	95% CI	β	95% CI
**Model 1: All patients**										
Dysnatremia vs. none	0.27	0.03–2.62	0.13	−3.02 to 10.6	−0.29*	−35.0–(−6.4)	0.26*	0.13–1.10	0.30*	0.40–1.82
Dyskalemia vs. none	5.00	0.36–69.1	0.29*	0.47–11.7	−0.01	−16.9–16.0	0.07	−0.40 to 0.72	0.01	−0.79 to 0.83
Dysnatremia plus dyskalemia vs. none	4.00	0.42–38.6	0.23*	1.89–6.86	−0.31*	−36.7–(−9.30)	0.23*	0.11–0.98	0.22*	0.13–1.52
AKI	9.16*	1.12–74.9	0.10	−1.44 to 7.34	0.02	−8.07 to 11.1	0.07	−0.17–0.47	0.01	−0.45–0.54
Delirium	1.94	0.53–7.12	0.09	−1.64–6.73	−0.21*	−23.8–(−5.55)	0.22*	0.18–0.79	0.27*	0.51–1.47
Hemoglobin	0.91	0.60–1.37	−0.43	−1.33 to 0.47	−0.11	−3.50–0.44	0.13	−0.004–0.13	0.12	−0.01–0.19
Diuretics	NA	NA	−0.02	−5.61 to 4.62	0.10	−2.88–18.6	−0.14*	−0.73–(−0.02)	−0.11	−1.01–0.11
**Model 2: Subgroup analysis**										
Resolution of dysnatremia	0.16*	0.14–0.63	−0.11	−8.98 to 2.54	0.07	−8.15 to 19.90	−0.06	−0.61 to 0.30	−0.02*	−0.87–(−0.65)
Resolution of dyskalemia	7.28	0.01–7145	−0.01	−7.43 to 6.89	0.36	−27.1 to 10.0	0.13	−0.33 to 1.05	0.11	−0.48 to 1.34
Resolution of dysnatremia plus dyskalemia	0.34	0.04–2.90	−0.06	−5.22 to 2.41	0.08	−5.48–16.5	−0.11	−0.60 to 0.11	−0.09	−0.90 to 0.21
Recovery of AKI	0.01*	0.001–0.14	−0.06	−6.66 to 2.77	0.11	−1.84 to 19.8	−0.20*	−0.91–(−0.20)	−0.22*	−1.59–(−0.45)

Model 1: adjusted for age, gender, BMI, Charlson comorbidity index, hemoglobin, white blood cell, eGFR, sodium, potassium, psychotropic agents, renin angiotensin system inhibitors, diuretics, delirium, AKI, dysnatremia vs. none, dyskalemia vs. none, dysnatremia plus dyskalemia vs. none.

Model 2: adjusted for age, gender, BMI, Charlson comorbidity index, hemoglobin, white blood cell, eGFR, dysnatremia, dyskalemia, sodium, potassium, psychotropic agents, renin angiotensin system inhibitors, diuretics, delirium, AKI, resolution of dysnatremia, resolution of dyskalemia, resolution of dysnatremia plus dyskalemia, recovery of AKI.

*AKI* acute kidney injury, *ADL* activities of daily living, *ECOG* eastern cooperative oncology group, *CFS* clinical frailty score, *LOS* length of stay.

*P < 0.05.

To further assess the impact of electrolyte and/or AKI recovery on hospital outcomes, individual subgroup analyses were performed in patients with electrolyte imbalance and AKI (Table 3, model 2). The results showed that resolution of dysnatremia was related to lower mortality risk and better CFS score. Recovery of AKI was also associated with a lower risk of mortality and better ECOG performance and CFS score.

## Discussion

This prospective study observed that acutely hospitalized elderly patients with initial electrolyte imbalance had higher percentages of AKI, higher in-hospital mortality rates, longer hospital stays, and worse ratings of the ADL scale, ECOG performance and CFS at discharge compared to those without imbalance. Multivariate analyses showed that dysnatremia and AKI predicted subsequent functional decline and mortality risk, respectively, and subgroup analyses revealed that recovery from dysnatremia and AKI were related to beneficial in-hospital outcomes. These findings highlight the importance of early detection and prompt intervention of both conditions during acute geriatric care.

In this study, we observed that patients with a higher CCI, lower BMI or lower hemoglobin were more likely to have a greater risk of dysnatremia. The findings were consistent with previous studies that showed higher CCI in patients with severe hyponatremia as well as lower BMI and hemoglobin in dysnatremic patients^3,19^. Most cases of dysnatremia were found at admission or during the initial 72 h of hospitalization. The patients mostly had low serum sodium levels, a finding which was analogous to the high prevalence of hyponatremia, as opposed to hypernatremia, noted among older hospital inpatients^3,21^. That being said, both conditions are equally strong predictors of increased hospital complications and mortality in elderly patients^22,23^. By contrast, the proportion of hypokalemia approximated that of hyperkalemia in our dyskalemic subgroups, which might explain the apparently ‘normal’ baseline potassium concentrations observed in these patients. Severe hypokalemia or hyperkalemia may lead to lethal cardiac events due to inherent arrhythmogenicity, if left untreated^24,25^ Furthermore, severe CKD tended to be more common in patients with dyskalemia, with or without dysnatremia, which was compatible with previous findings that hyperkalemia is one of the most common and lethal electrolyte disorders in CKD^26^. Therefore, all elderly inpatients should be monitored for the evolution of electrolyte profiles, particularly dysnatremia and dyskalemia, and treated accordingly to prevent adverse hospital outcomes. Additionally, we also observed that patients with initial electrolyte imbalance were associated with longer hospital stays. This finding is consistent with previous observations that hospitalized patients with dysnatremia or dyskalemia exhibited longer LOS^27–29^, which reiterates the importance of maintaining electrolyte balance during the course of hospitalization.

The distribution of mortality rates in this cohort exhibited a U-shaped relation to initial serum sodium and, to a lesser extent, serum potassium concentrations. Although this phenomenon has been demonstrated in hospitalized or critically ill patients with dysnatremia and dyskalemia^6,7,14,19,30,31^, it has rarely been reported in an acute geriatric setting^32^. Furthermore, patients with initial dysnatremia plus dyskalemia whose condition did not resolve displayed the highest mortality rate, compared to those who recovered from the abnormalities (Fig. 2A). In addition to in-hospital mortality, dysnatremia, regardless of potassium levels, was associated with worse performance of the ADL scale, ECOG score and CFS at discharge. More importantly, our subgroup analyses confirmed that recovery from dysnatremia (in dysnatremic patients) was associated with reduced mortality risk and improved CFS score. Other researchers have also reported that resolution of hyponatremia has a beneficial impact on geriatric function as assessed by the ADL scale and the Mini-Mental State Examination^17^. It is worth mentioning that our study did not observe initial dysnatremia or dyskalemia alone was independently associated with mortality after hospital admission^6,7,31^. Instead, we found that restoration of electrolyte abnormalities during acute geriatric care could serve as a beneficial indicator for in-hospital outcomes. The reason for this discrepancy was not completely clear but could be related to distinct phenotypes of studied populations or dynamic nature of electrolyte balancing. Suffice to say that for hospitalized elderly patients every effort should be made to correct electrolyte imbalances regardless of the reason alongside the treatments for the acute medical conditions.

AKI is frequently encountered in acutely hospitalized elderly patients. Prior studies have demonstrated that patients with AKI are more likely to have concomitant electrolyte imbalance, particularly dysnatremia and dyskalemia^33^, and the presence of AKI at admission is associated with reduced functional capacity as assessed by the ADL scale^34^. Similarly, we observed that patients with electrolyte imbalance showed higher percentages of AKI, which independently predicted the risk of in-hospital mortality in our model. Furthermore, recovery from AKI was related to reduced mortality risk and improved ECOG performance and CFS score. Intriguingly, patients with AKI superimposed on dysnatremia or dyskalemia presented with a higher mortality rate than those with isolated AKI (Fig. 2B), suggesting that the complexity of electrolyte imbalance may aggravate the negative impact of AKI in relation to hospital outcomes.

Almost all usage of diuretics was started at the emergency room while waiting to be admitted. This finding is reminiscent of a prior cross-sectional report that showed that diuretic treatment at the emergency department was significantly associated with dysnatremia and dyskalemia. The use of loop diuretics was more likely associated with hypernatremia and hypokalemia, whereas thiazide diuretics was an independent risk factor for hyponatremia and hypokalemia^35^. As mentioned earlier, these disorders are potent predictors of increased morbidity or mortality in acute elderly inpatients and should be avoided whenever possible. Conversely, we observed that diuretic use was associated with better ECOG performance at discharge, and a significant proportion of diuretic users were patients with acute decompensated heart failure, underlying congestive heart failure and coronary artery disease (75%, 48.6%, and 36.7%, respectively). These results, which are consistent with previous studies that showed diuretic usage could improve cardiac functional status^36,37^, support the contention that functional status and possibly in-hospital outcomes in elderly people with cardiac edema could be enhanced by prudent use of diuretic agents without incurring electrolyte imbalance.

Delirium is yet another complication frequently observed in elderly hospitalized patients and plays a crucial role in shaping the outcomes^38^. In this study, although no significant differences existed in the percentages of delirium among the four groups, we observed an increasing trend in the prevalence of delirium in patients with electrolyte imbalance. In addition, delirium strongly correlated with worse ratings on the ADL scale, ECOG performance and CFS at discharge, which is reminiscent of others’ findings that delirium is a risk factor for decreased instrumental ADL in geriatric patients after surgery^39^. These results underscore the importance of delirium prevention and management during acute geriatric care.

The merit of the present study is the demonstration that recovery from initial electrolyte imbalance and renal impairment was related to subsequent prognosis in a real-world geriatric ward setting. The tools used to evaluate functional status, i.e., ADL scale, ECOG performance and CFS, have been widely used to track changes in function and serve as outcome predictors during distinct acute settings^40–42^. However, this study may be subject to some limitations. For example, we did not distinguish the temporal course of electrolyte imbalance which would be pivotal in determining the rate of correction. Nevertheless, our protocol for dysnatremia management is to correct slowly, at a rate less than 0.5 mmol/L/h and no inadvertent cerebral complications had ever occurred during the course. One might also argue that the beneficial effects observed in this study were short-term at best. However, one study shows the continuous benefits of restoring electrolyte imbalance postdischarge^43^, thus extending the generalizability of the results accrued from this study.

In summary, for acutely hospitalized older patients, our data show that resolution of dysnatremia was associated with a lower risk of mortality and improved CFS rating, whereas recovery from AKI was associated with lower mortality risk and better ECOG performance and CFS score. These results suggest that restoration of initial dysnatremia and AKI during acute geriatric care may be instrumental in improving in-hospital survival and functional status at discharge.

## Methods

### Study design

From April 1, 2018 to November 30, 2018, we consecutively collected health-care data from elderly patients (≥ 65 years) who were admitted to an acute geriatric ward of the National Taiwan University Hospital. Patients were excluded if they were institutionalized before the index hospitalization or readmitted after the index hospitalization. The study was approved by the Research and Ethics Committee of the National Taiwan University Hospital (No. 201801099RINA) and followed principles outlined in the Declaration of Helsinki. Patient’s informed consent were obtained at admission. Baseline clinical data, including demographic characteristics, concurrent illnesses, and prescribed drugs were recorded at presentation. The CCI was calculated to evaluate the severity of comorbid conditions. Laboratory data were collected through hospitalization. The glomerular filtration rate was estimated by the isotope dilution mass spectrometry-traceable 4-variable Modification of Diet in Renal Disease Study equation. Functional scores on the Barthel scale of ADL, ECOG performance and CFS were assessed upon admission and prior to discharge by the primary care team (geriatrician and nurse staff). Baseline scores for ECOG performance and CFS were assigned via inquiry into patients’ conditions before admission, whereas those for the ADL scale represented the status at admission.

Upon admission or during the first 72 h of hospitalization, the diagnoses of AKI were made according to the serum creatinine criteria of the Kidney Disease Improving Global Outcomes classification^44^, and dysnatremia or dyskalemia were defined as a serum sodium or potassium level outside the physiologic range of 135–145 mmol/L and 3.5–5.0 mmol/L, respectively.

### Outcome measures

The main outcome measures were LOS, all-cause mortality, and functional status changes during hospitalization. The latter was assessed by calculating the differences in the scores of the ADL scale, ECOG performance, and CFS upon admission and before discharge. To compare the association of different electrolyte disorders on outcomes, we categorized patients into those with both dysnatremia and dyskalemia, those with dysnatremia alone, those with dyskalemia alone, and those without dysnatremia or dyskalemia. Electrolyte imbalance was deemed resolved when serum sodium or potassium levels returned to the normal range before discharge. Renal function was judged to recover when serum creatinine levels returned to within 0.3 mg/dl above baseline^44,45^. The baseline renal function was determined by acquiring the serum creatinine data three to six months prior to the index hospitalization. The resolution of electrolyte disorder and renal function recovery were judged according to the last blood sampling before hospital discharge.

### Statistical analysis

SAS software (version 9.4) was used for statistical analyses and Supplementary Figure S2. Figure 1, 2 and Supplementary Fig. S1 were carried out using the GraphPad Prism software (version 6.0). Continuous variables were described as the mean ± standard deviation with a normal distribution or median (interquartile range) for those with a skewed distribution. For numerical data, the Wilcoxon signed rank test was used for comparisons within groups, and the Kruskal–Wallis test was used for comparisons among the four groups of electrolyte imbalance. Categorical variables were presented as event numbers with percentages (%), and the chi-squared or Fisher’s exact test was used. The Pearson correlation coefficient was used to measure the correlation between serum sodium and potassium levels at admission.

Univariate and multivariate logistic regression models were constructed to investigate the predictors of all-cause mortality. Additionally, univariate and multivariate linear regressions were performed to identify the predictors of LOS and functional status changes during hospitalization. Variables that were significant in the univariate analysis were included in the multivariate model. Covariates adjusted in the regression models included participants’ clinical and laboratory data, as well as states of dysnatremia, dyskalemia, and AKI. A two-sided *P* < 0.05 was considered statistically significant.

## Supplementary Information

Supplementary Information.

## References

[1] Hoorn EJ, Zietse R (2013). Hyponatremia and mortality: Moving beyond associations. Am. J. Kidney Dis..

[2] Snyder NA, Feigal DW, Arieff AI (1987). Hypernatremia in elderly patients. A heterogeneous, morbid, and iatrogenic entity. Ann. Intern. Med..

[3] Hu J (2017). Dysnatremia is an independent indicator of mortality in hospitalized patients. Med. Sci. Monit..

[4] Lu DY (2016). Hyponatremia and worsening sodium levels are associated with long-term outcome in patients hospitalized for acute heart failure. J. Am. Heart Assoc..

[5] Mannesse CK (2013). Prevalence of hyponatremia on geriatric wards compared to other settings over four decades: A systematic review. Ageing Res. Rev..

[6] Wald R, Jaber BL, Price LL, Upadhyay A, Madias NE (2010). Impact of hospital-associated hyponatremia on selected outcomes. Arch. Intern. Med..

[7] Waikar SS, Mount DB, Curhan GC (2009). Mortality after hospitalization with mild, moderate, and severe hyponatremia. Am. J. Med..

[8] Adrogué HJ, Madias NE (2000). Hypernatremia. N. Engl. J. Med..

[9] Himmelstein DU, Jones AA, Woolhandler S (1983). Hypernatremic dehydration in nursing home patients: An indicator of neglect. J. Am. Geriatr. Soc..

[10] Rabinstein AA, Wijdicks EF (2003). Hyponatremia in critically ill neurological patients. Neurologist.

[11] Mc Causland FR, Wright J, Waikar SS (2014). Association of serum sodium with morbidity and mortality in hospitalized patients undergoing major orthopedic surgery. J. Hosp. Med..

[12] Lindner G, Pfortmüller CA, Leichtle AB, Fiedler GM, Exadaktylos AK (2014). Age-related variety in electrolyte levels and prevalence of dysnatremias and dyskalemias in patients presenting to the emergency department. Gerontology.

[13] Tongyoo S, Viarasilpa T, Permpikul C (2018). Serum potassium levels and outcomes in critically ill patients in the medical intensive care unit. J. Int. Med. Res..

[14] Bouadma L (2019). Influence of dyskalemia at admission and early dyskalemia correction on survival and cardiac events of critically ill patients. Crit. Care (London, England).

[15] Miller AJ (2017). Dysnatremia in relation to frailty and age in community-dwelling adults in the national health and nutrition examination survey. J. Gerontol. Ser. A Biol. Sci. Med. Sci..

[16] Suárez V (2020). Impairment of neurocognitive functioning, motor performance, and mood stability in hospitalized patients with euvolemic moderate and profound hyponatremia. Am. J. Med..

[17] Brinkkoetter PT (2019). Impact of resolution of hyponatremia on neurocognitive and motor performance in geriatric patients. Sci. Rep..

[18] Darmon M (2014). Influence of early dysnatremia correction on survival of critically ill patients. Shock.

[19] Han SS (2016). Dysnatremia, its correction, and mortality in patients undergoing continuous renal replacement therapy: A prospective observational study. BMC Nephrol..

[20] Chawla A, Sterns RH, Nigwekar SU, Cappuccio JD (2011). Mortality and serum sodium: Do patients die from or with hyponatremia?. Clin. J. Am. Soc. Nephrol. (CJASN).

[21] Mohan S, Gu S, Parikh A, Radhakrishnan J (2013). Prevalence of hyponatremia and association with mortality: Results from NHANES. Am. J. Med..

[22] Imai N, Sumi H, Shibagaki Y (2019). Impact of age on the seasonal prevalence of hypernatremia in the emergency department: A single-center study. Int. J. Emerg. Med..

[23] Naka T, Kohagura K, Kochi M, Ohya Y (2018). Hyponatremia and mortality among very elderly residents in a geriatric health service facility. Clin. Exp. Nephrol..

[24] Unwin RJ, Luft FC, Shirley DG (2011). Pathophysiology and management of hypokalemia: A clinical perspective. Nat. Rev. Nephrol..

[25] Rossignol P (2016). Emergency management of severe hyperkalemia: Guideline for best practice and opportunities for the future. Pharmacol. Res..

[26] Dhondup T, Qian Q (2017). Electrolyte and acid-base disorders in chronic kidney disease and end-stage kidney failure. Blood Purif..

[27] Betts KA (2018). the cost of hyperkalemia in the United States. Kidney Int. Rep..

[28] Corona G (2016). The economic burden of hyponatremia: Systematic review and meta-analysis. Am. J. Med..

[29] Felizardo Lopes I, Dezelée S, Brault D, Steichen O (2015). Prevalence, risk factors and prognosis of hypernatraemia during hospitalisation in internal medicine. Netherlands J. Med..

[30] Sakr Y (2013). Fluctuations in serum sodium level are associated with an increased risk of death in surgical ICU patients. Crit. Care Med..

[31] Matsushita K (2019). Dyskalemia, its patterns, and prognosis among patients with incident heart failure: A nationwide study of US veterans. PLoS ONE.

[32] Correia L (2014). Severe hyponatremia in older patients at admission in an internal medicine department. Arch. Gerontol. Geriatr..

[33] Chao CT (2015). The severity of initial acute kidney injury at admission of geriatric patients significantly correlates with subsequent in-hospital complications. Sci. Rep..

[34] Chao CT (2015). Cross-sectional study of the association between functional status and acute kidney injury in geriatric patients. BMC Nephrol..

[35] Arampatzis S (2013). Impact of diuretic therapy-associated electrolyte disorders present on admission to the emergency department: a cross-sectional analysis. BMC Med..

[36] Peacock WF (2009). Impact of intravenous loop diuretics on outcomes of patients hospitalized with acute decompensated heart failure: Insights from the ADHERE registry. Cardiology.

[37] Murray MD (2001). Open-label randomized trial of torsemide compared with furosemide therapy for patients with heart failure. Am. J. Med..

[38] GMR, C (2019). Delirium is prevalent in older hospital inpatients and associated with adverse outcomes: Results of a prospective multi-centre study on World Delirium Awareness Day. BMC Med..

[39] Liang CK (2014). Interrelationship of postoperative delirium and cognitive impairment and their impact on the functional status in older patients undergoing orthopaedic surgery: A prospective cohort study. PLoS ONE.

[40] Fortinsky RH, Covinsky KE, Palmer RM, Landefeld CS (1999). Effects of functional status changes before and during hospitalization on nursing home admission of older adults. J. Gerontol. Ser. B Biol. Sci. Med. Sci..

[41] Shohei Asada AD, Ueno H, Murata K, Yanase K (2020). Yoshiji, H Efficacy and safety of ERCP in elderly patients with an ECOG performance status of 3–4. World Acad. Sci. J..

[42] Theou O (2020). Exploring clinically meaningful changes for the frailty index in a longitudinal cohort of hospitalized older patients. J. Gerontol. Ser. A Biol. Sci. Med. Sci..

[43] Corona G (2015). Hyponatremia improvement is associated with a reduced risk of mortality: Evidence from a meta-analysis. PLoS ONE.

[44] Okusa MD, Davenport A (2014). Reading between the (guide)lines–The KDIGO practice guideline on acute kidney injury in the individual patient. Kidney Int..

[45] Chou YH (2017). Renin-angiotensin system inhibitor is associated with lower risk of ensuing chronic kidney disease after functional recovery from acute kidney injury. Sci. Rep..

