# Supplementary material for: Restoration of dysnatremia and acute kidney injury benefits outcomes of acute geriatric inpatients
Source: Sci Rep. 2021 Oct 11;11:20097. doi: 10.1038/s41598-021-99677-z (PMC8505420; doi:10.1038/s41598-021-99677-z)

Table S1. Outcomes of the study participants with or without dysnatremia and dyskalemia during hospitalization

|  | All  (n=401) | Dysnatremia plus dyskalemia  (n=130) | Dysnatremia alone  (n=114) | Dyskalemia alone  (n=59) | None  (n=98) | *P* value |
| --- | --- | --- | --- | --- | --- | --- |
| **Events during hospitalization** | | | | | | |
| AKI, total, n (%)*  AKI, stage 1, n (%)^#^  AKI, stage 2, n (%)^#^  AKI, stage 3, n (%)^#^ | 184 (46)  129 (70)  29 (16)  26 (14) | 68 (52)  44 (65)  10 (15)  14 (20) | 50 (44)  35 (70)  11 (22)  4 (8) | 34 (58)  24 (70)  5 (15)  5 (15) | 32 (33)  26 (82)  3 (9)  3 (9) | 0.01  0.41  0.47  0.21 |
| Delirium, n (%) | 153 (38) | 57 (44) | 39 (35) | 27 (46) | 30 (31) | 0.15 |
| Length of stay*, days (SD) | 12.7 (10.9) | 14.8 (11.1) | 12.73 (13.4) | 14.0 (11.1) | 9.2 (5.4) | < 0.01 |
| In-hospital mortality, n (%) | 18 (5) | 7 (5) | 7 (6) | 3 (5) | 1 (1) | 0.42 |
| **Restoration of electrolyte imbalance and AKI** | | | | | | |
| Dysnatremia resolution, n (%) | 175 (72) | 91 (70) | 84 (74) | -- | -- | 0.59 |
| Dyskalemia resolution, n (%) | 137 (73) | 91 (70) | -- | 46 (78) | -- | 0.26 |
| AKI recovery, n (%) | 143 (77) | 49 (71) | 40 (80) | 25 (74) | 29 (91) | 0.15 |

**P* < 0.05

AKI, acute kidney injury; SD, standard deviation

Figure S1. The percentages of outcome improvement in different categories of serum sodium and potassium levels at admission. (a) Activities of Daily Living (ADL), (b) Eastern Cooperative Oncology Group (ECOG) performance, (c) Clinical Frailty Scale (CSF), (d) Length of stay. Figures were created using the GraphPad Prism software (version 6.0).Figure S2. Correlations of serum sodium and potassium levels at admission. Figures were created using SAS software (version 9.4).


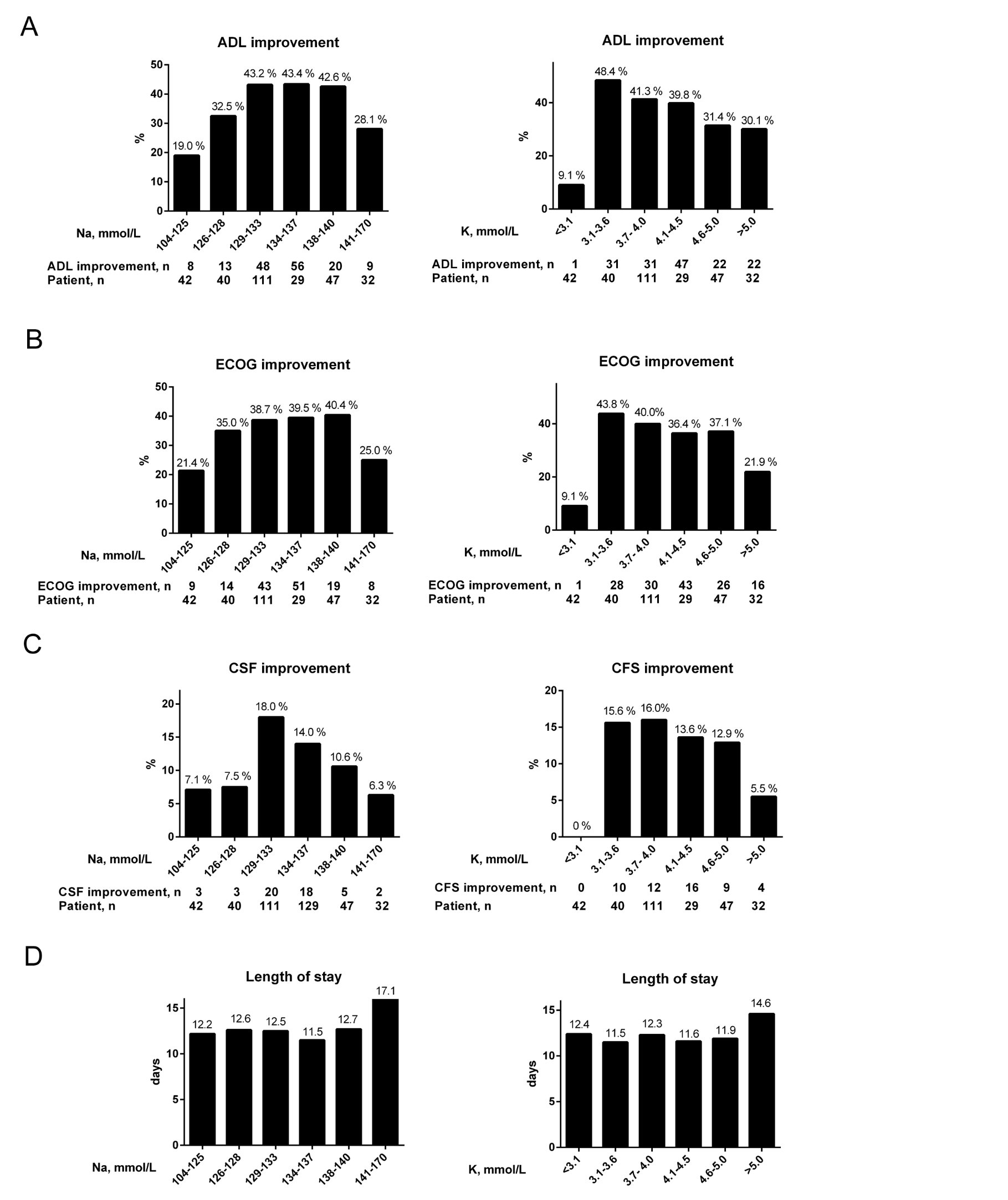


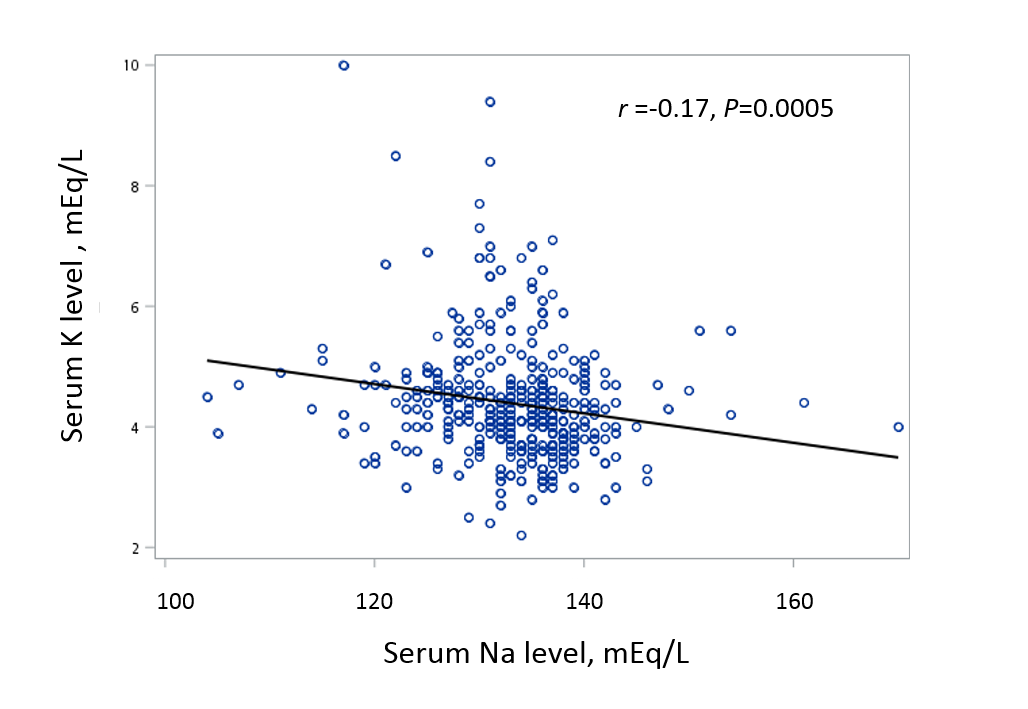

Supplement: Supplementary file 1 — Supplementary Information. [file 41598_2021_99677_MOESM1_ESM.docx]
